# Occlusive wound dressings: A greenhouse for bacteria?

**DOI:** 10.1177/17571774241261923

**Published:** 2024-06-15

**Authors:** Vendela M Scheer, Johan H Scheer, Anders Kalén, Lena Serrander

**Affiliations:** 1 Department of Biomedical and Clinical Sciences, 59591 Linköping University, Linköping, Sweden; 2 Department of Orthopedics, Department of Biomedical and Clinical Sciences, 59591 Linkoping University Faculty of Medicine, Linköping, Sweden; 3 Division of Clinical Microbiology, Department of Biomedical and Clinical Sciences, 59591 Linköping University, Linköping, Sweden

**Keywords:** Wound dressing, occlusive dressings, post-operative dressings, bacteria recolonisation

## Abstract

**Background:**

The modern wound dressing is produced to absorb fluid and protect against external contamination. The choice of which wound dressing to apply after surgery is usually based on local tradition. There are various impervious dressings on the market. Even if the wound is sterile before application, there will be subsequent recolonisation of skin microbiota. Previous studies suggest that a high bacterial load on the skin hampers wound healing and might be a risk for SSI.

**Aim:**

The aim was to compare bacterial recolonisation on the shoulder under three different wound dressings, 48 h after sterile preparation of the skin as in preparation for surgery.

**Method:**

In 25 healthy volunteers, a standard pre-surgical skin disinfection for a deltopectoral incision was made on the left shoulder with 0.5% chlorhexidine solution in 70% ethanol. Three different wound dressings were then placed on the shoulder, and 48 h later the skin beneath each dressing was swabbed, subsequently cultured and bacterial density analysed using viable count.

**Results:**

The bacterial recolonisation under air-dry (gauze) dressing was significantly lower (*p* = .0001) compared to semipermeable and occlusive wound dressings.

**Conclusion:**

Choosing a less permeable wound dressing may lead to an increased bacterial load on the skin during the first 48 h after surgery.

## Introduction

Surgical Site Infection (SSI) is perhaps the most serious complication a patient can encounter after surgery. The etiology of SSI is multifactorial. Surgical environment, surgical technique and patient susceptibility to infection contributes (Atesok et al., 2017). Safeguarding the patient from SSI can be divided into three phases, pre-, intra- and postoperative. The importance of decreased bacterial burden on the skin before and during surgery has been well studied since Joseph Lister in the late 1900s experimented with different kinds of disinfection techniques (Toledo-Pereyra, 2010). It has also been shown that bacterial load with over 10^5^ CFU/mLof certain bacteria is a risk factor for wound infection (Turtiainen et al., 2014).

In primary healing, the wound is re-epithelialized between 24 and 72 h (Chrintz et al., 1989; Schoukens, 2009). If a wound is critically colonized, the increasing number of bacteria might become a bioburden and thereby delay wound healing (Sood et al., 2014) or even be a cause of an SSI (Sandy-Hodgetts et al., 2022; Turtiainen et al., 2014).

After closure of a surgical incision, a dressing is applied, the function of which is to absorb wound fluid and prevent external contamination (Obagi et al., 2019). The history of how to protect and promote wound healing goes back to more than 2000 years BC. Herbs were used for healing, honey for preventing and curing infection and raw meat was used to stop bleeding (Sipos et al., 2004). The methods and materials of today’s wound dressings differ, but the purpose is still the same. From treating wound with dry dressings to development of dressings according to the principle of moist wound healing (Nuutila and Eriksson, 2021), the market in the 80s was catapulted into a new era (Jones, 2005; Queen et al., 2004). Today, the health care professionals have a plethora of products available. Clinical evidence supporting the choice of a dressing is hard to find (Dhivya et al., 2015; Yuan et al., 2022). There is a lack of high-quality research evidence whether any wound dressing is better than others (Borkar and Khubalkar, 2011; Dumville et al., 2016; Tan et al., 2020). Many studies are made with support of the manufacturing companies themselves, with an obvious risk of bias (Arroyo et al., 2015; Dumville et al., 2014).

### Different dressings

It is not easy to get an overview of the market and its divisions of different wound dressings. One way is to divide them according to permeability:

• Dry dressings/Permeable dressings: Gauze dressings are made by woven or non-woven fibres of cotton, rayon or polyester. They are designed for absorbing discharge from the wound.
• Semipermeable dressings: they are designed and marketed as being impermeable to external fluid and bacteria, but permeable to internal air and water vapour in order to reduce the risk of maceration (Jones et al., 2006).
• Occlusive dressings: are thought to improve healing by keeping the wound moist (Pickles et al., 2022).

*Cutibacterium acnes (C.acnes),* is a gram positive facultative anaerobic bacterium and a human commencal that resides predominantly in the piloeosebaceous glands of the skin. These glands are more abundant in the forehead, shoulder, thorax and lower back (Achermann et al., 2014). It is well documented that *C.acnes* is the predominant bacterium causing SSI after shoulder surgery (Grosso et al., 2018; Levy Pierre et al., 2008; Nelson et al., 2016). In our previous studies, we have investigated how to reduce the bacterial burden on the skin before and during shoulder surgery, focussing on *C. acnes* (Scheer et al., 2018, 2021b). In this study we focus on the postoperative phase. This non-surgical study aimed to compare bacterial recolonisation, under three different wound dressings, 48 h after sterile skin disinfection of the shoulder, using the same culturing protocol as in our previous published work on skin disinfection in shoulder surgery (Scheer et al., 2018, 2021a, 2021b).

## Methods

**Figure 1.**
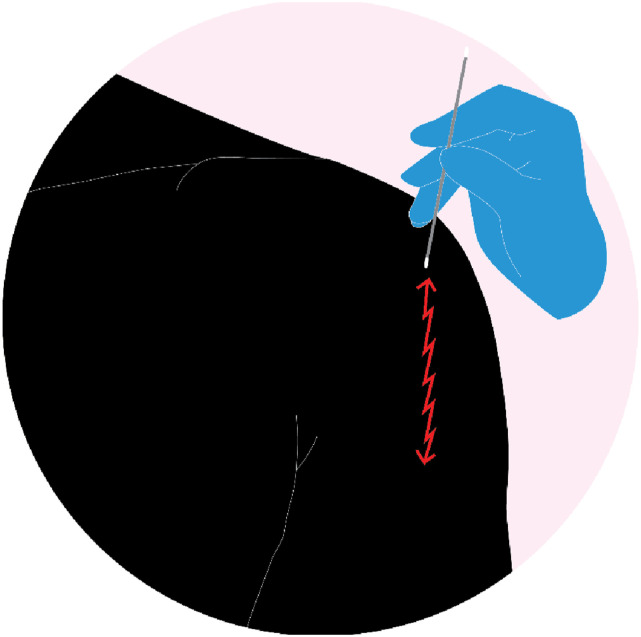
PES-technique, rub the swab with an oscillating movement – like using a pencil eraser – going down over a 10 cm line, then in the same manner up again for a total of 15 passages.

Men have a higher amount of *C. acnes* on the shoulder than women. To optimize for *C. acnes,* we included men only (Dizay et al., 2017; Patel et al., 2009; Scheer et al., 2021b). Twenty-five healthy male volunteers aged 28–65 (mean 46 years), gave informed consent to participate. Inclusion criteria were being male, age > 18 years with legal capacity. Exclusion criteria were any visible skin lesion in the shoulder area or any antimicrobial treatment – oral or local – within 7 days of the experiment (to maximise bacterial count). Four skin swabs were collected – as described below – from the left shoulder using the Pencil Eraser Swab (PES) technique. A swab is rubbed with an oscillating movement – like using a pencil eraser – going down over a 10 cm line and then in the same manner up again for a total of 15 times (Figure 1). The technique is designed to maximise harvest of bacteria, without skin abrasion (Scheer et al., 2021a). We used an eSwab (Copan Italia S.p.A. via Perotti 10, Brescia, Italy), a flocked swab with a tube, containing 1 mL of liquid Amies, which elutes the entire sample into the medium.

**Figure 2.**
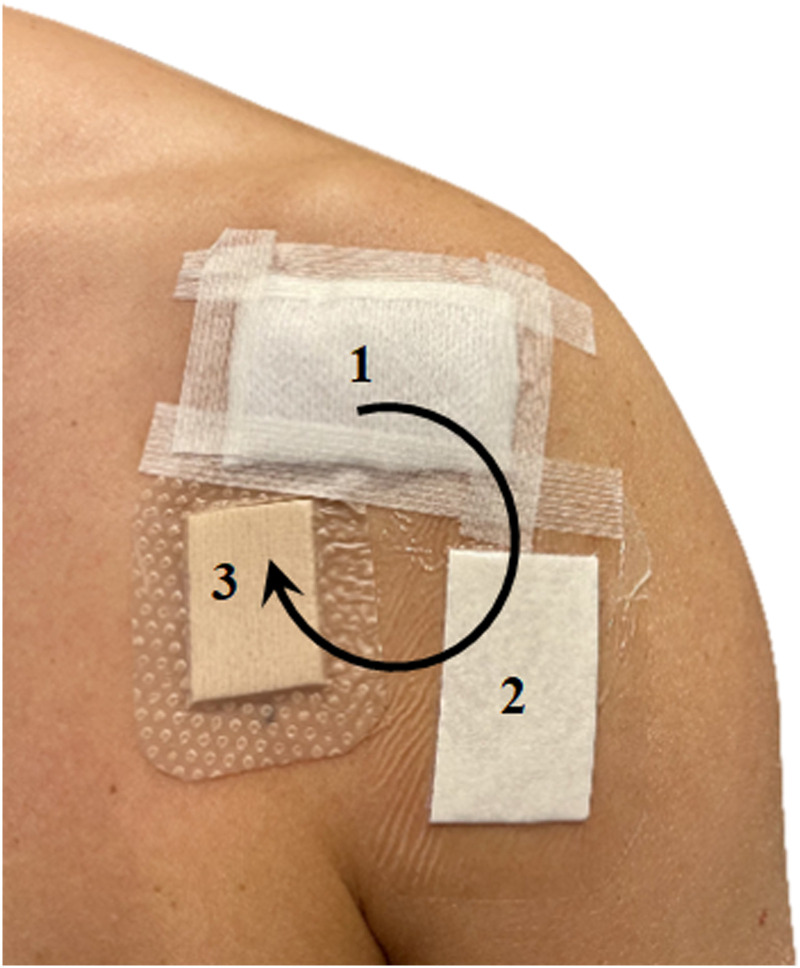
1: Gauze, 2: Tegaderm Pad film dressing^®^. 3: Mepilex Border Lite^®^. The dressings were moved one position clockwise for the next study subject.

On the left shoulder, before skin preparation, the first skin swab was collected, which served as a control. Thereafter the same shoulder was the scrubbed for 2 min with 0.5% chlorhexidine solution in 70% ethanol and then allowed to dry, as would have been done in preparation for surgery, before three different types of wound dressings were placed on the shoulder. The dressings were moved one position clockwise on the next subject with the three dressings always covering the same three areas (Figure 2).

### Dressing descriptions

1. Gauze: 100 % Cotton, 4 layers 5 × 5 cm (Mölnlycke Health Care, Gothenburg, Sweden)
2. Occlusive dressing: Tegaderm Pad film dressing® 5 × 5 cm (3M, Maplewood, USA)
3. Semipermeable film dressing: Mepilex Border Lite® 4 × 5 cm (Mölnlycke Health Care, Gothenburg, Sweden)

After approximately 48 h the wound dressings were removed, one at a time, and the skin beneath each wound dressing was swabbed using the PES-technique.

### Microbiological technique

All skin swabs were immediately put into the medium and cold storage before being transported to the laboratory within 12 h. After vortexing for 10 s, a serial dilution was made (1:10, 1:100, 1:1000) and thereafter cultivated for 5 days on anaerobic antibiotic-free agar plates in an anaerobic incubator at 36^o^. The colony forming units (CFU) were counted and viable count (VC) is expressed as CFU/mL (Ben-David and Davidson, 2014). Matrix-assisted laser desorption/ionisation time-of-flight mass spectrometry (MALDI-Tof) were used for determination of bacterial species.

### Statistical analyses

Wilcoxon’s nonparametric test for Related Samples was used to compare viable counts.

## Results

The control swab, taken before skin preparation, displayed a wide range of bacterial count as well as in the three other groups (Table 1). The dominating species identified was *C.acnes,* followed by *Staphylococcus epidermidis*. Some other Staphylococci species were also identified, *S. saccharolyticus, S. Caprae* and *S. Capitis.* When comparing to what extent bacteria regrew under different dressings for 48 h, there was no difference between the control swab and semipermeable dressing (*p* = .619) nor between control and occlusive dressing (*p* = 7716). The only significant difference was found between control and airy dressing (*p* = .0001). Bacterial growth was significantly lower under the airy dressing when compared to semipermeable (*p* = .009) and occlusive (*p* = .017) dressings. However, there was no difference between semipermeable and occlusive count (*p* = .414).

**Table 1.** Viable Count in Groups.

Median	Control	Semipermeable	Occlusive	Airy
Median viable count (CFU/mL) (Range)	13 300	3 900	4 500	260
	[130 – 400000]	[0 – 410000]	[0 – 1960000]	[0 – 200000]

Semipermeable: Mepilex Border Lite® Occlusive: Tegaderm Pad film dressing® Airy: Gauze.

## Discussion

The results showed that the bacterial recolonisation under an airy dressing was significantly lower after 48 h than after more occlusive dressings. Lower bacterial load during this vulnerable phase is desirable from wound healing perspective (Turtiainen et al., 2014).

Thanks to the Human Microbiome Project, we know that even though individuals have similar core microbiome, there are variations at different body sites (Murray et al., 2021). This is reflected on SSIs in respective area and depending on where, different bacteria are predominant in causing infections. For example, in hip surgery, Coagulase-negative Staphylococci (CoNS) is the most common bacterium identified (Lindgren et al., 2014). Correspondingly *C. acnes* is in shoulder surgery (Nelson et al., 2016; Richards et al., 2014). Sampling skin microbiome may produce different results depending on the technique used. The PES-technique used in the current study was developed to evaluate different skin treatments before shoulder surgery (Scheer et al., 2021a). The incubation in an anaerobic environment is probably to *C. acnes* advantage. Even if CoNS grows under anaerobic circumstances, the balance in between these bacteria could have been different using another culturing regimen.

Wound dressings after surgery have many purposes, one of them being to protect from cross contamination of the environment and hands (exogenous contamination) (Dhivya et al., 2015). The plethora of wound dressings available on the market today, and the lack of evidence results in local choice of dressing after surgery often becomes an in-hospital tradition (Sood et al., 2014). The manufacturers of semipermeable wound dressings and occlusive dressings claim that wound healing is promoted by moisture (Jones, 2005). In wound healing by primary intention, the surgical wound is epithelialized after 48 h. The crust provides a seal and protect the wound from contamination and optimizes the wound healing (Schoukens, 2009). A negative effect of semipermeable or occlusive wound dressing might be the risk of skin maceration, which can lead to a loss of barrier function and increases the risk of infection (Kevin et al., 2017)

We do not know when bioburden on the skin becomes critical. Krizek et al. (Krizek et al., 1975) performed a study on skin grafts, and a critical point appeared to be when the bacterial count was over 10^5^ CFU/mL but the relationship between dose of bacterial contamination and the resilience of the patient may be the determining factor (Namba et al., 2013; Pulido et al., 2008). Another aspect regarding orthopaedic patients is that orthopaedic surgery is more sensitive to infection, due to surgery involving joints and often implicate implantation of large amounts of foreign material. In a rabbit model, Southwood et al. concluded that less than 50 CFU of *S. aureus* seeded at the time of surgery was enough to cause an infection (Southwood et al., 1985).

High bacterial burden in surgical wounds may be detrimental for wound healing and might lead to an SSI (Sandy-Hodgetts et al., 2022). Turtiainen et al. found that a high bacterial load in surgical lower limb wounds on the second postoperative day significantly increased the risk for an SSI, as an independent risk factor. Furthermore, studies with antimicrobial dressings reducing the bacterial content on the surface and that in itself reduces wound infections (Grosso et al., 2017; Whitley et al., 2023). These, however, carry the inherent risk of development of bacterial resistance in a wider population (Ousey, 2023*)*, for example, with silver dressings (Terzioglu et al., 2022).

### Limitations

We have not measured any other characteristics for an ideal dressing, such as minimal pain during removal, comfort for the host to wear, excess exudates. This study enrolled healthy volunteers and did only include intact skin. The bacterial recolonisation might differ from a surgically closed wound. Also, there is not much high level evidence for the association between bacterial burden and SSI.

## Conclusion

In the Operating Theatre, pre- and peri-operative focus is safeguarding the patient from Surgical Site Infections (SSI) both from exogenous and endogenous factors. An approach that should follow the patient until the wound has healed.

The recolonisation under airy dry wound dressing is significantly lower than under semipermeable and occlusive wound dressing in the shoulder area. To protect the wound from being inoculated by bacteria, primary closure must be done properly. Hopefully this study will constitute a possible basis for future studies, evaluating different wound dressings, including comparison of the bacterial burden beneath with the overall purpose to decrease the risk of SSIs.
